# Cereulide and Deoxynivalenol Increase LC3 Protein Levels in HepG2 Liver Cells

**DOI:** 10.3390/toxins14020151

**Published:** 2022-02-18

**Authors:** Julia Beisl, Gudrun Pahlke, Monika Ehling-Schulz, Giorgia Del Favero, Doris Marko

**Affiliations:** 1 Department of Food Chemistry and Toxicology, Faculty of Chemistry, University of Vienna, Währinger Straße 38, 1090 Vienna, Austria; julia.beisl@univie.ac.at (J.B.); gudrun.pahlke@univie.ac.at (G.P.); 2 Department of Pathobiology, Institute of Microbiology, University of Veterinary Medicine, Veterinärplatz 1, 1210 Vienna, Austria; monika.ehling-schulz@vetmeduni.ac.at; 3 Core Facility Multimodal Imaging, Faculty of Chemistry, University of Vienna, Währinger Straße 38, 1090 Vienna, Austria

**Keywords:** mitophagy, *Fusarium*, *Bacillus cereus*, hepatotoxicity, mold

## Abstract

Food contaminants of bacterial or fungal origin frequently contaminate staple foods to various extents. Among others, the bacterial toxin cereulide (CER) and the mycotoxin deoxynivalenol (DON) co-occur in a mixed diet and are absorbed by the human body. Both toxins exert dis-tinctive mitotoxic potential. As damaged mitochondria are removed via autophagy, mitochondrial and lysosomal toxicity were assessed by applying low doses of single and combined toxins (CER 0.1–50 ng/mL; DON 0.01–5 µg/mL) to HepG2 liver cells. In addition to cytotoxicity assays, RT-qPCR was performed to investigate genes involved in lysosomal biogenesis and autophagy. CER and DON caused significant cytotoxicity on HepG2 cells after 5 and 24 h over a broad concentration range. CER, alone and in combination with DON, increased the transcription of the autophagy related genes coding for the microtubule associated protein 1A/1B light chain 3 (*LC3)* and sequestome 1 (*SQSTM1)* as well as LC3 protein expression which was determined using immunocytochemistry. DON increased LC3 protein expression without induction of gene transcription, hence it seems plausible that CER and DON act on different pathways. The results support the hypothesis that CER induces autophagy via the LC3 pathway and damaged mitochondria are therefore eliminated.

## 1. Introduction

Food spoilage or contamination by mycotoxins or bacterial toxins is a global problem. Ingested toxins might affect several organ systems ranging from local manifestations limited to the gastrointestinal tract to broad systemic distribution. As the prime organ of biotransformation, bioactivation and excretion the liver is often targeted by food contaminants. Hence, it is the primary site of aflatoxin B1 toxicity, but also of other fungal and bacterial metabolites such as the mycotoxin deoxynivalenol (DON) and the bacterial toxin cereulide (CER) which frequently occur in staple foods in various concentrations [1,2,3,4]. CER, a cyclic dodecadepsipeptide ([D-O-Leu-D-Ala-L-O-Val-L-Val]_3_) [5,6], produced by some genetically related *Bacillus cereus* strains [7], was described to cause acute liver failure [8,9] and diffuse microvesicular steatosis as well as necrosis [10,11]. DON, a *Fusarium* mycotoxin ubiquitously contaminating grains, leads to diverse and partly contradictory effects on liver cells including cytotoxicity or reduced albumin secretion [12,13,14].

The potassium ionophore CER was described to cause mitochondrial swelling [15] and impairment of mitochondrial respiration [16]. Furthermore, DON is associated with various pathophysiological responses and was reported to decrease mitochondrial membrane potential and area/mass as well as to down-regulate mitochondrial respiratory chain elements and import proteins [17,18,19,20]. 

In order to remove damaged organelles, increased degradation/turnover via autophagy is necessary [21]. In this process, a phagophore is built around the designated cargo, subsequently forming an autophagosome. After fusion with a lysosome, an autolysosome is generated and the content is degraded by lysosomal hydrolases [21]. In addition to general autophagy, selective macroautophagy targeting mitochondria, namely mitophagy takes place. To ensure specificity, adaptor proteins such as sequestosome 1 (SQSTM1 or p62), which regulate selective macroautophagy, are interacting with membrane bound microtubule-associated protein 1A/1B-light chain (LC3) [22]. LC3 is furthermore necessary for autophagosomal biogenesis and is synthesized in a higher amount when autophagy is induced [21,22]. Different proteins involved in autophagy have been described to be regulated on a transcriptional level and have been associated with the Coordinated Lysosomal Expression and Regulation (CLEAR) motif [23,24]. Furthermore, autophagy is influenced by several transcription factors and signaling cascades such as transcription factor EB (TFEB), mammalian target of rapamycin (mTOR) or nuclear factor kappa B (NFκB) [25,26].

While DON was already reported to cause an increase in LC3 protein in HT-29 intestinal cells [27] and to interfere with lysosomal trafficking [28], reports on autophagy related effects of CER are still lacking. We recently reported CER and DON to cross the membrane barrier of an intestinal cell model [29]. Hence, it seems realistic that also the lipophilic toxin CER reaches the liver when administered in low doses and could therefore exert hepatotoxic effects. Furthermore, CER was detected in various tissue samples from piglets fed with CER including liver, intestine and adipose tissue [30]. Due to the mitochondrial damaging potential of CER, we hypothesized that both substances may have an impact on lysosomes as well as autophagy-related pathways in liver cells. Therefore, the aim of the study was to investigate the substances’ effect alone and in combination on lysosomal biogenesis and autophagy induction.

In this manuscript we show that CER increased *MAPLC3B* and *SQSTM1* gene transcription and LC3 protein expression alone and in combination with DON hence supporting the hypothesis that CER induces autophagy via the LC3 pathway. Even though DON by itself possesses the ability to increase the LC3 protein content, the lack of an increased *MAPLC3B* and *SQSTM1* gene transcription indicates a distinct mechanism of action.

## 2. Results

### 2.1. Cell Viability

**Figure 1:**
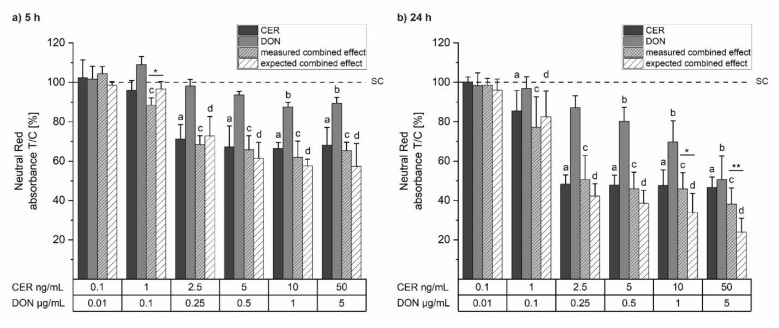
Cell viability of HepG2 cells after (**a**) 5 h and (**b**) 24 h incubation with cereulide (CER; dark grey), deoxynivalenol (DON; grey), a combination of both toxins (measured combined effect; light grey, dashed), a calculated combination (expected combined effect; white, dashed) measured by Neutral Red assay. Results are presented as means + standard deviations, related to the solvent control (SC; 1% *v/v* dimethyl-sulfoxide (DMSO), 1% *v/v* H_2_O; dashed line) (*n* ≥ 4). The expected combined effects were determined by Independent Joint Action, for details see Section 5.9, Equation (1). Significant differences to the respective no-effect level were calculated by one-way ANOVA (*p <* 0.05) followed by Bonferroni post-hoc test and are indicated as “a” (CER), “b” (DON), “c” (measured combined effect) and “d” (expected combined effect). Significant differences between the measured and the expected combined effect, indicated with “*” (*p* < 0.05) and “**” (*p* < 0.01), were calculated by Student’s *t*-test.

To evaluate the cytotoxic potential of CER, DON and their combination on HepG2 cells and to ensure the use of non-cytotoxic concentrations in the subsequent assays, cell viability was monitored after 5 h and 24 h. All used substances reduced cell viability depending on their concentration and the incubation time (Figure 1). After 5 h incubation, both CER alone and in combination with DON led to a reduction of cell viability to about 65–70% starting from 2.5 ng/mL CER with or without 0.25 µg/mL DON (Figure 1a). On the other hand, DON showed minor cytotoxic effects, reducing the cell viability to approximately 90% in the highest concentration applied (5 µg/mL DON). After 24 h of incubation, cell viability was significantly reduced by all substances to levels of 46%, 50% and 38 % by CER, DON or their combination, respectively (Figure 1b). The onset of cytotoxicity was achieved by lower concentrations after a prolonged incubation time of 24 h. Furthermore, co-incubation with CER and DON showed antagonistic cytotoxic behavior in the two highest tested concentrations after 24 h of incubation.

### 2.2. MitoTracker^®^

**Figure 2:**
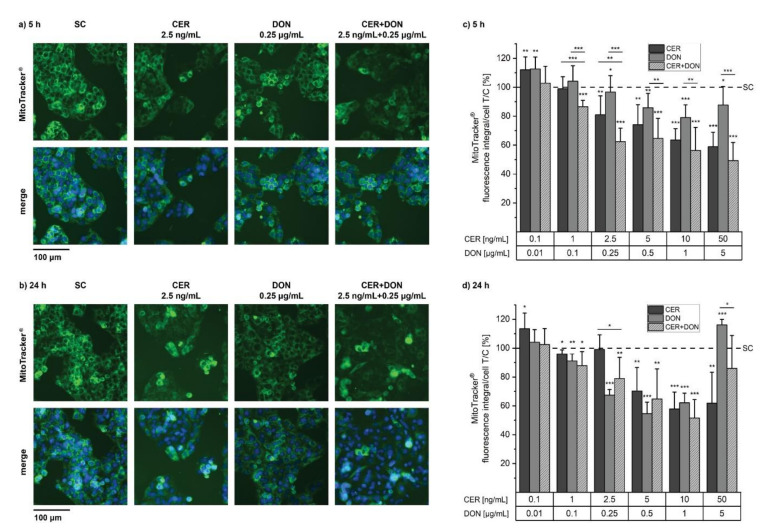
Mitochondria of HepG2 cells were labelled with MitoTracker^®^ Green FM after 5 h (**a**,**c**) and 24 h (**b**,**d**) incubation with CER, DON or the respective combination. (**a,b**)show example images after 5 h and 24 h, (**c**,**d**) depict the quantitative analysis of mitochondria per cell after incubation with CER (dark grey), DON (grey) or their combination (light grey, dashed). Results of at least 5 biological replicates are depicted as means + standard deviations, related to the solvent control (SC, 1% *v/v* DMSO, 1% *v/v* H_2_O; dashed line). Significant differences between the compounds and the solvent control were assessed with one-sample Student’s *t*-test. All significances are indicated with “*” (*p* < 0.05) “**” (*p* < 0.01) and “***” (*p* < 0.001).

As CER and DON are both known to damage mitochondria, MitoTracker experiments were performed after 5 h (Figure 2a,b) and 24 h (Figure 2c,d) in the same concentrations as cell viability assays. Even though CER and DON had no effect on cell viability in the lowest concentration of 0.1 ng/mL and 0.01 µg/mL, respectively, an increased number of mitochondria was detected after 5 h for both single substances and after 24 h with CER. With increasing concentrations, the number of mitochondria decreased at both time points with one exception: 24 h incubation with the highest concentration of 5 µg/mL DON alone or in combination with 50 ng/mL CER led to an increase of mitochondria suggesting possible artefacts as a result of pronounced cytotoxicity (representative images in Supplementary Materials, Figure S1). In general, the quantitative analysis is representative of the acquired images. Nevertheless, a closer look at the images of 24 h incubation with CER revealed mitochondrial aberrations at a concentration of 2.5 µg/mL even though the fluorescence signal is still at the level of the solvent control (Figure 2b,d). Due to opposing effects on mitochondria, mathematical modelling to predict synergism and antagonism was not possible as it was for cell viability assays. However, the combination of CER and DON already in low concentrations (CER:DON, 1 ng/mL:0.1 µg/mL) resulted in a significant and concentration-dependent decline of mitochondria after 5 h whereas their appearance was not impaired by the single compounds, thus suggesting a synergistic effect (Figure 2a,c). The combination of CER and DON led to a significant reduction of mitochondria compared to DON alone over a broad concentration range after 5 h. However, these effects did not persist after 24 h possibly due to the pronounced loss of cell viability through both substances after prolonged incubation.

### 2.3. LysoTracker^®^

**Figure 3:**
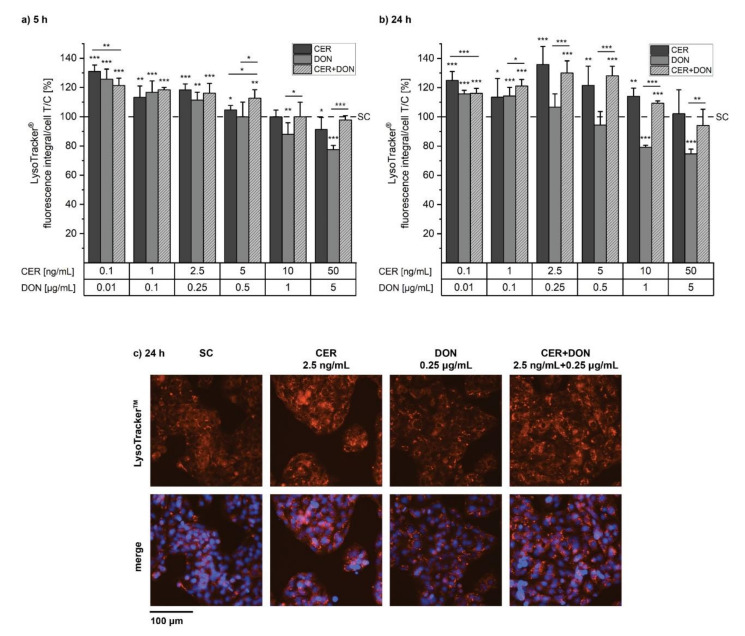
Lysosomal changes were monitored with LysoTracker^®^ Red DND-99. HepG2 cells were incubated with CER (dark grey), DON (grey) or the respective combination (light grey, dashed) for (**a**) 5 h or (**b**) 24 h. Data of at least 5 biological replicates is presented as means + standard deviations, related to the solvent control (SC, 1% *v/v* DMSO, 1% *v/v* H_2_O; dashed line). Significant differences between the compounds and the solvent control were assessed with one-sample Student’s *t*-test. Significant differences between the single substances and the respective combinations were determined with two-sample Student’s *t*-test and significances are indicated with “*” (*p* < 0.05) “**” (*p* < 0.01) and “***” (*p* < 0.001). (**c**) Representative images after 24 h of incubation with solvent, 2.5 ng/mL CER, 0.25 µg/mL DON or the combination of both.

As mitochondria are commonly degraded by lysosomes, the number of lysosomes was monitored by performing LysoTracker^®^ experiments simultaneously to MitoTracker^®^. After 5 h, CER significantly increased the number/size of lysosomes over a broad concentration range of 0.1–5 ng/mL (Figure 3a). Finally, at a concentration of 50 ng/mL CER the lysosomes were reduced to 91 % of the solvent control. Similar to CER, DON also enhanced the number/size of the lysosomes, with the difference that the lysosomes started to decrease at lower concentrations, dropping to approximately 77% at a concentration of 5 µg/mL even though cytotoxic effects were less pronounced at this concentration compared to CER. The combination of CER and DON showed a similar response as CER alone. 24 h incubations with CER/DON led to similar effects in the two lowest used concentrations compared to 5 h experiments (Figure 3b). Additionally, after 24 h, 2.5 ng/mL CER significantly increased the lysosomal signal up to 136%. In general, the effect of the substance combinations on lysosomes appears to be dominated by CER. Figure 3c shows representative images of fluorescence microscopy used for quantification of data as depicted in Figure 3a,b.

### 2.4. LysoSensor^TM^

**Figure 4:**
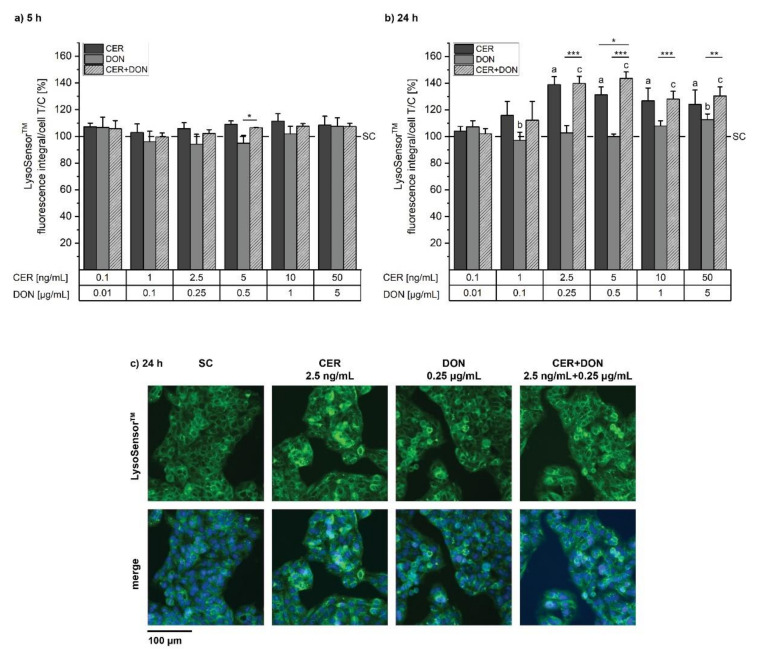
Quantitative analysis of acidic lysosomes after (**a**) 5 h and (**b**) 24 h incubation with CER (dark grey), DON (grey) and the respective combination (light grey, dashed) investigated with LysoSensor^TM^ Green DND-189. Results are presented as means + standard deviations, normalized to the solvent control (SC, 1% *v/v* DMSO, 1% *v/v* H_2_O; dashed line) (*n* ≥ 4). Significant differences to the respective no-effect level were calculated by one-way ANOVA (*p <* 0.05) followed by Bonferroni post-hoc test and are indicated as “a” (CER), “b” (DON) and “c” (measured combined effect). Significant differences between the single substances and the respective combination, indicated with “*” (*p <* 0.05), “**” (*p* < 0.01) and “***” (*p* < 0.001), were calculated by two-sample Student’s *t*-test. (**c**) depicts examples of acquired images after 24 h incubation with solvent, 2.5 ng/mL CER, 0.25 µg/mL DON or the combination of both.

To further evaluate alterations in lysosomal function, LysoSensor^TM^ experiments were performed allowing to detect lysosomal acidification. According to the manufacturer, the used probe is only fluorescent in acidic compartments, allowing the quantification of acidified lysosomes and therefore indicating proper function. In contrast to LysoTracker^®^ results, LysoSensor^TM^ results revealed no significant effects on lysosomal function after 5 h of incubation in any of the experimental conditions (Figure 4a). Nevertheless, after 24 h of incubation both CER and the respective combination with DON significantly increased the fluorescence signal up to around 140 % of solvent control starting from 2.5 ng/mL CER with or without 0.25 µg/mL DON (Figure 4b). DON alone also significantly increased acidified lysosomes at the highest tested concentration of 5 µg/mL, thus showing a contrary effect compared to LysoTracker^®^. In this data set the effect on the lysosomes also appears to be driven by CER. The images clearly show the induction of the fluorescence signal (Figure 4c).

### 2.5. Gene Transcription Analysis: Quantitative Real-Time PCR (qPCR)

**Figure 5:**
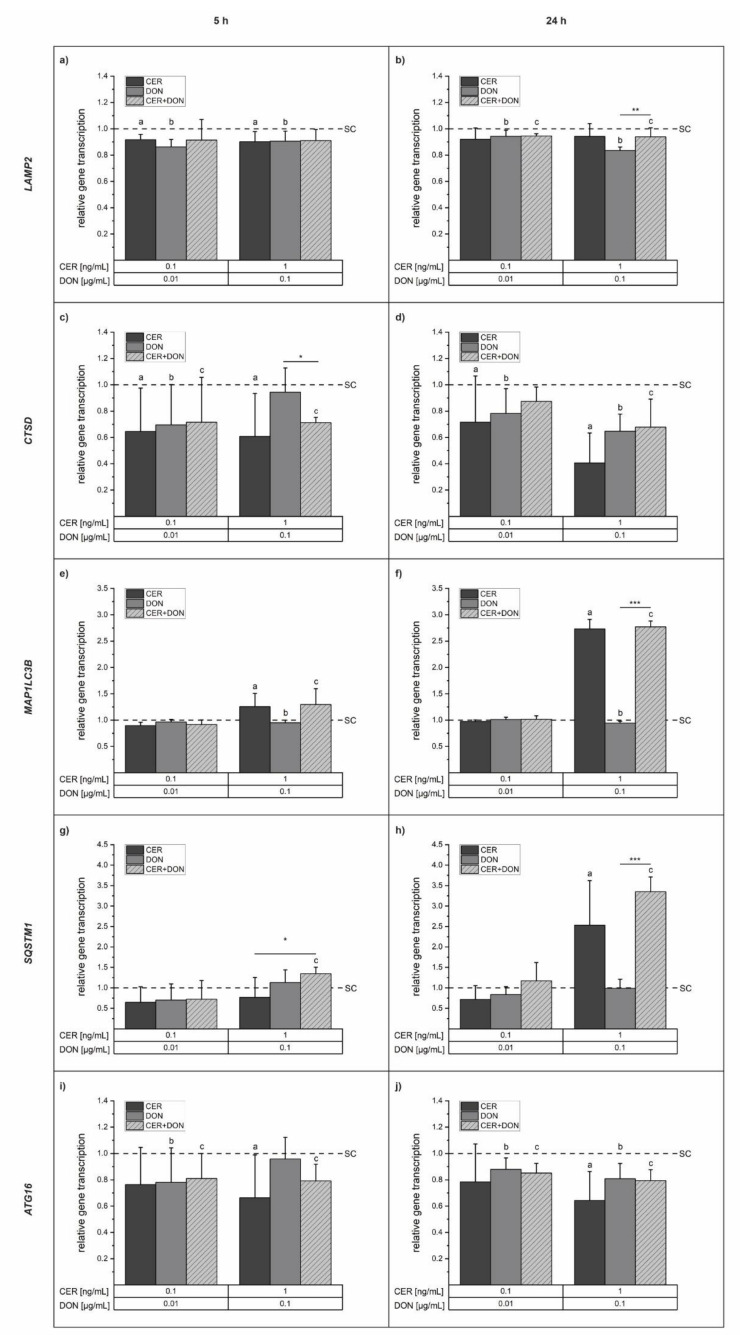
Fold change of mRNA transcript levels of *LAMP2*, *CTSD*, *MAP1LC3B*, *SQSTM1*, and *ATG16L1* after 5 h (**a**,**c**,**e**,**g**,**i**) and 24 h (**b**,**d**,**f**,**h**,**j**) incubation of HepG2 cells with CER (dark grey), DON (light grey) or the combination of both (light grey, dashed). mRNA levels were assessed with qRT-PCR, calculated by the 2^−ΔΔCt^ method [31] and normalized to the solvent control (SC, dashed line) as fold change. All target genes were related to the endogenous control genes *HPRT1* and *ALAS1*. Results are depicted as means + standard deviations of at least four biological replicates. Significant differences to the solvent control were calculated by one-way ANOVA (*p <* 0.05) followed by Bonferroni post-hoc test and are indicated as “a” (CER), “b” (DON) and “c” (measured combined effect). Significant differences between the single substances and the respective combinations were analyzed with Student’s *t*-test and are indicated with “*” (*p* < 0.05), “**” (*p* < 0.01) or “***” (*p* < 0.001).

As lysosomes were significantly affected by CER, we further investigated the impact on the transcription of genes involved in lysosomal structure and biogenesis. To prevent possible artefacts due to cytotoxicity, the two lowest concentrations of CER (0.1 and 1 ng/mL) and DON (0.01 and 0.1 µg/mL) were chosen for further experiments. Concerning lysosomal structural components, transcriptional analysis of the lysosome-associated membrane glycoprotein 2 (*LAMP2*; Figure 5a,b) and the lysosomal protease cathepsin D (*CTSD*; Figure 5c,d) was performed. Single compounds as well as their combinations marginally reduced *LAMP2* mRNA levels with statistical significance after 5 h as well as after 24 h incubation however, suggesting no major effect on *LAMP2* as gene transcription did not drop below 0.5-fold. The transcription of *CTSD* was reduced in a more pronounced way and dropped below 0.5-fold for the incubation with 1 ng/mL CER after 24 h (Figure 5c,d). Furthermore, we investigated the gene transcription of the autophagy related genes *MAP1LC3B* (Figure 5e,f), *SQSTM1* (Figure 5g,h) and *ATG16* (Figure 5i,j). While 0.1 µg/mL DON led to a slight but statistically significant reduction of *MAP1LC3B* gene transcription at both time points, 1 ng/mL CER alone and in combination with 0.1 µg/mL DON increased transcript levels after 5 h and 24 h reaching an approximately 2.7-fold induction after 24 h. An elevation of more than 2-fold of mRNA suggests pronounced changes in gene transcription. *SQSTM1* was increased 2.5-fold and 3.3-fold after 24 h incubation with 1 ng/mL CER alone or in combination with 0.1 µg/mL DON, respectively. After 5 h incubation, a rise in *SQSTM1* mRNA levels by the respective combination became already apparent. The third autophagy related gene, *ATG16* was reduced significantly, though only to a minor extent by all test substances at different concentrations and time points.

### 2.6. Immunofluorescence Analysis of LC3

**Figure 6:**
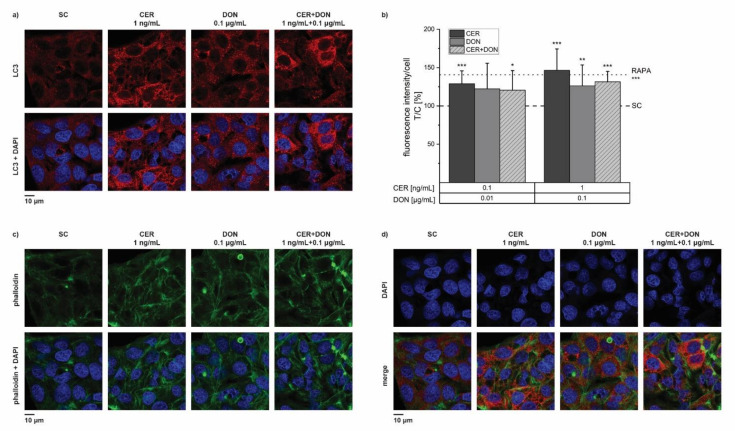
Immunofluorescence staining and quantitative analysis of LC3 and phalloidin after 24 h incubation of HepG2 cells with CER, DON, their combination, rapamycin (RAPA; 100 nM; positive control) or solvent control (SC). The microscopy panels (**a**,**c**,**d**) depict the single channel pictures of LC3, phalloidin or DAPI (4′,6-diamidino-2-phenylindole) in the upper row and the merge pictures with the nuclei (stained with DAPI) in the lower row. The bar chart (**b**) represents the quantitative analysis of the LC3 fluorescence intensities after incubation with CER (dark grey), DON (grey) and a combination of both (light grey, dashed). Results are presented as means + standard deviation of 3 biological replicates including in total of at least 11 optical fields and are normalized to the solvent control (dashed line). Rapamycin (RAPA) as a positive control is indicated as the dotted line. Significant differences between the solvent control and the treated samples were calculated by one-sample Student’s *t*-test. All significances are indicated with “*” (*p* < 0.05), “**” (*p* < 0.01) or “***” (*p* < 0.001).

Due to the induction of *MAP1LC3B* gene transcription by CER after 24 h incubation we further investigated the protein expression of LC3 with an immunofluorescence staining. Results clearly show an induction of LC3 after 24 h incubation with CER and DON, both alone and in combination (Figure 6a). Quantitative analysis of fluorescence intensities of LC3 per cell revealed a significant rise of LC3 protein by all tested substances and concentrations except 0.01 µg/mL DON (Figure 6b). Rapamycin (RAPA), a known autophagy inducer served as a positive control in all biological replicates. As expected, RAPA significantly induced LC3 to 140% of the solvent control. CER in the concentration of 1ng/mL reached a similar level with 146% indicating the induction of autophagy via the LC3 pathway. Furthermore, staining of the cytoskeletal actin filaments was performed with phalloidin. The cytoskeleton of HepG2 cells showed no abnormalities potentially induced by the different substance concentrations (Figure 6c). Strikingly, we observed changes in nuclear morphology, namely deformation and loss of roundness, of cells treated with the higher concentration of CER, especially when combined with DON (Figure 6).

## 3. Discussion

In this study, we aimed at elucidating the effects of CER and DON, alone and in combination, on mitochondria and lysosomes including autophagy and mitophagy. For the first time, we report that CER increased LC3 gene expression and protein content in HepG2 liver cells, suggesting an increased formation of autophagosomes. The applied substance concentrations are based on occurrence and exposure data as previously described in detail [29]. Furthermore, CER was shown to be able to pass an in vitro intestinal epithelium of differentiated Caco-2 cells [29]. Hence, CER is likely to reach systemic circulation and also the human liver as it was already described in piglets and was suggested to be bioavailable due to its detection in adipose tissue and different organs [30].

Both CER and DON decreased the signal intensity of stained mitochondria, which is indicative of mitochondrial toxicity in HepG2 cells over a broad concentration and time range in our experiments (Figure 2). This is in line with literature, as DON was already reported to disorganize the mitochondrial network in a concentration and time dependent way in A431 cells [19] and to permeabilize mitochondrial membranes in both HCT116 intestinal cells and HepG2 liver cells [17,18]. Furthermore, the effects of CER on mitochondria of various cell lines were described to be quite pronounced and included swelling or disruption of inner membranes [15,32].

Damaged mitochondria are known to be removed via autophagy and therefore engulfed by lysosomes. Hence, we hypothesized that the observed mitochondrial damage may influence lysosomal function or biogenesis as they are connected via the CLEAR gene network [23]. Indeed, LysoTracker^®^ and LysoSensor^TM^ experiments showed an increase in measured signal indicating an increased number and size of lysosomes (Figure 3). The fusion of lysosomes with autophagosomes and hence a higher volume in acidic organelles may be the reason for this signal. Furthermore, Ivanova, et al. [33] reported that the mycotoxin Enniatin B led to increased lysosomal size prior to lysosomal disintegration together with the loss of mitochondrial membrane potential in intestinal cells. Being ionophores such as CER, it cannot be excluded that, to some extent, Enniatins may exert similar toxic effects. Concerning DON, Del Favero, et al. [28] reported accumulation of lysosomes in the perinuclear region as well as decreased lysosomal movement after DON incubation: this was attributed by alterations of cytoskeletal elements necessary for vesicular transport.

As lysosomal number and size can only be an indicator for a variety of processes, further investigations regarding the elucidation of responsible mechanisms were carried out. Conducting qPCR experiments, we investigated the expression of genes involved in lysosomal biogenesis and autophagy. Of note, LAMP2 plays an important role in the progression of autophagy as it is involved in the regulation of autophagosome-lysosome fusion and *LAMP2* deficiency may lead to impaired autophagy [34]. The genes coding for the lysosomal membrane protein LAMP2 and the lysosomal hydrolase cathepsin D, previously associated with lysosomal biogenesis [35], were marginally affected by CER and DON (Figure 5) suggesting a mechanism other than pure biogenesis involved in the cells’ response to CER.

The autophagy related genes *MAP1LC3B* and *SQSTM1* coding for LC3 and p62 protein, respectively [26], showed significantly increased transcript levels after 24 h incubation with CER while unaffected by DON. SQSTM1/p62 is recruited to mitochondria to prime them for degradation [36] and its expression is increased as a result of mitophagy [37]. Interestingly, Rakovic, et al. [38] reported different mitochondrial depolarizing agents namely carbonyl cyanide-4-(trifluoromethoxy) phenylhydrazone (FCCP) and valinomycin, a structure analogue of CER [5], to target different mitophagy pathways. After valinomycin treatment, damaged mitochondria were removed by the ubiquitin proteasome system and not through autophagy via the LC3 related pathway [38].

To further investigate the impact of CER on mitophagy, we additionally determined LC3 on the protein level using an immunofluorescence approach. Indeed, the level of LC3 protein was increased by CER, DON and the combination as well as the positive control rapamycin. Cho, et al. [39] showed that the autophagy inducer rapamycin attenuated liver injury caused by chronic alcohol exposure. The same kind of liver injury was characterized by the impairment of the autophagic flux [39], raising the question whether the observed effects caused by CER may be attributed to either the impairment of autophagic flux or increased autophagosome formation. Steady state methods for investigating autophagy including gene and protein expression analysis of *SQSTM1*, *MAPLC3B* and LC3 facilitate the assessment of the formation of autophagic organelles [40], which is reflected in the obtained data. However, the applied methods do not permit commenting on autophagic flux or accumulation of autophagic organelles [40].

Recently, low micromolar concentrations of DON were reported to increase LC3 protein content in HT-29 intestinal cells [27]. Here we received similar results for HepG2 liver cells. Contrary to CER, DON does not induce *MAPLC3B* or *SQSTM1* gene expression, hence it is tempting to speculate that various molecular pathways are involved in the autophagic response to the toxins. This hypothesis is substantiated by recent results by Del Favero, et al. [19] reporting DON induced reduction of several proteins involved in ubiquitination and the proteasome complex. Hence, we hypothesize that DON impairs the autophagic flux rather than induce autophagy. However, Di Malta, et al. [25] reported several transcription factors to be involved in autophagy, stressing the complexity of the process.

## 4. Conclusions

In conclusion, for the first time we present evidence that CER induces autophagy via the LC3 pathway. Mitochondria damaged by CER seem to be degraded by lysosomes, leading to an increased number of lysosomes as well as the induction of the autophagy related genes *SQSTM1* and *MAPLC3B*. Even though DON by itself possesses the ability to increase the LC3 protein content, different mechanisms of action seem to be involved. Interestingly, in most measured endpoints, CER seems to be the dominating compound. As CER crosses the intestinal barrier and is able to reach various tissues [29,30], further studies regarding its autophagy altering effect are recommended as medical conditions, such as Parkinson’s disease or ethanol induced liver injury are associated with defective autophagy.

## 5. Materials and Methods

### 5.1. Chemicals and Materials

DON was purchased from Romer Labs (Tulln, Austria; 99.4% purity) while CER was purified according to Bauer, et al. [30]. Identity and purity of CER was determined by LC-MS/MS and 1D/2D NMR as previously described in Bauer, et al. [41] resulting in a purity of 98%. Roswell Park Memorial Institute (RPMI) 1640 cell culture medium and supplements were obtained from Gibco^®^ Life Technologies (Karlsruhe, Germany). Glycine, formaldehyde and Triton X-100 were purchased from Carl Roth (Karlsruhe, Germany). Neutral Red was obtained from Sigma-Aldrich (Taufkirchen, Germany).

### 5.2. Cell Culture: HepG2 Cell Line

HepG2 cells, a widely used liver cell line established from hepatocellular carcinoma, were acquired from the Leibniz Institute German Collection of Microorganisms and Cell Cultures (DMSZ, Braunschweig, Germany) and were used for all experiments. HepG2 growth medium consisted of RPMI 1640 medium containing 10% (*v*/*v*) fetal calf serum (FCS) and 100 U/mL penicillin, 100 µg/mL streptomycin. Cells were cultivated under humidified conditions (37 °C, 5% CO_2_, 95% humidity). Sub-cultivation was performed 2–3 times/week at a cell density of 80–90%. Cells were seeded for experiments in 96-well plates, 24 well plates or µ-Slide 8 Well ibiTreat (ibidi, Gräfelfing, Germany). Cell passages used for experiments ranged from 10 to 25 and mycoplasma contamination of the cells was routinely monitored.

### 5.3. Incubation Conditions and Dose Selection

For stock solutions, CER was dissolved in dimethyl sulfoxide (DMSO) and DON in water and solvent control samples contained equal amounts of both solvents. HepG2 cells were treated with 0.1–50 ng/mL CER (corresponding to 0.09–43.4 nM CER) and/or 0.01–5 µg/mL DON (corresponding to 0.03–16.9 µM DON) or a constant ratio of CER to DON of 1:100 with a final DMSO concentration of 1% (*v/v*). The dose selection is based on occurrence data [1,2,3,4,42,43] as well as previous results including intestinal uptake experiments [29,30]. The incubation conditions comprise a broad concentration range to reflect varying contamination levels as previously described in detail in Beisl, et al. [29] and it was assumed that the substances were fully bio-accessible and diluted in 1 L gastric fluid [44,45]. Results were obtained from at least three biological replicates per assay.

### 5.4. Cell Viability: Neutral Red Assay

The Neutral Red (NR) Assay was performed as previously described [46,47]. Briefly, 10,000 cells/well were seeded in 96-well plates and allowed to grow for 48 h prior to incubation. Thereafter, cells were incubated with various concentrations of CER, DON or a combination of both for 5 h, 24 h. All plates included solvent and positive controls (0.1% (*v/v*) Triton-X 100). NR dye was dissolved in Dulbecco’s phosphate buffered saline (DPBS) to reach a stock concentration of 4 mg/mL. The day before use, the NR stock solution was further diluted to 40 µg/mL in cell culture medium (NR medium) and incubated at 37 °C. To remove undissolved dye crystals, NR medium was centrifuged for 10 min at 600× *g* and subsequently filtered with filter paper. After the respective time, incubation medium was replaced by NR medium and incubated for 3 h before washing with DPBS to remove unbound dye. Cells were then treated with 150 µL of de-staining solution (50:50:1 ethanol absolute, dH_2_O, glacial acetic acid) and shaken for 10 min at 500 rpm on a plate shaker. After transferring 130 µL of de-staining solution to a fresh 96-well plate, absorbance was measured at 540 nm using a Cytation3 imaging reader (BioTek, Winooski, VT, USA). Results are related to the respective solvent control (test/control [%]).

### 5.5. MitoTracker^®^ and LysoTracker^®^

The number of mitochondria and lysosomes was monitored by the simultaneous staining of living HepG2 cells with MitoTracker^®^ Green FM and LysoTracker^®^ Red DND-99 (Molecular Probes by life technologies, Eugene, OR, USA) adapting a previously used protocol [28]. 12,000 or 10,000 HepG2 cells/well were seeded in 96-well plates (clear bottom, black side), allowed to grow for two days and subsequently incubated with CER, DON or in combination for 5 h or 24 h, respectively. Cells were then stained with growth medium containing 1 µM MitoTracker^®^ and 1 µM LysoTracker^®^ and 10 µg/mL Hoechst 33258 for 30 min at 37 °C. After washing the cells two times with growth medium, cells were covered with growth medium containing 0.25 µM LysoTracker^®^ to prevent a loss of fluorescent signal and cell blebbing according to the manufacturer’s manual. The analysis was performed by automatic acquisition of pictures with a Cytation3 imaging reader (BioTek, Winooski, VT, USA) equipped with a 20× magnification objective as well as GFP (469/525), Texas Red (586/647) and DAPI (377/447) filter cubes. Prior to calculations, blurred pictures were removed resulting in a total of 6–12 pictures per condition and biological replicate. Image analysis was performed with Gen5^TM^ 3.08 software (BioTek, Winooski, VT, USA) applying dual mask image analysis as previously reported in Held [48]. Briefly, fluorescence thresholds were defined for all fluorescence channels (GFP, Texas Red and DAPI) to enable the correct detection of the nuclei and acidified lysosomes present in the cytosol. Hence, the fluorescence integral (fluorescence intensity normalized to the area) per cell were calculated. Results were further normalized to the solvent control (test/control [%]).

### 5.6. LysoSensor^TM^

To investigate the acidification of lysosomes, HepG2 cells were stained with LysoSensor^TM^ Green DND-189 (Molecular Probes by life technologies, Eugene, OR, USA) as the dye accumulates in lysosomes resulting in an increased fluorescence intensity upon acidification. The assay was performed in the same way as described in chapter 5.5 with minor adaptions. Briefly, cells were incubated with 50 µL of growth medium containing a final concentration of 1 µM LysoSensor^TM^ as well as 10 µg/mL Hoechst 33258 for 30 min., followed by washing the wells with 100 µL of growth medium. For imaging, cells were covered with 100 µL of growth medium containing 0.25 µM LysoSensor^TM^ to prevent a loss of fluorescent signal and cell blebbing according to the manufacturer’s protocol. Pictures were automatically acquired with a Cytation3 imaging reader (BioTek, Winooski, VT, USA) using a 20× magnification objective and GFP (469/525) and DAPI (377/447) filter cubes. For analysis, blurred pictures were excluded from analysis resulting in a total of 8–12 optical fields per biological replicate. Image analysis was performed as described in 5.5.

### 5.7. Gene Transcription Analysis: Quantitative Real-Time PCR

Changes in gene transcription of genes associated with lysosomal structure and biogenesis as well as autophagy were investigated with quantitative real-time PCR (qPCR). After 5 h and 24 h of incubation with CER and DON, extraction of total RNA was performed with a Maxwell^®^ 16 LEV simplyRNA Cells Kit (Promega, Madison, WI, USA) according to the manufacturer’s protocol. Briefly, cells were washed with PBS, followed by the addition of 200 µL of chilled homogenization solution containing thioglycerol. An equal amount of lysis buffer was added to the homogenized cells and the samples were vortexed for 15 s prior transferring them into the Maxwell^®^ 16 LEV simplyRNA cartridges. Furthermore, DNAse I was added to the respective cartridge well before automatic RNA extraction. Concentration and purity of the obtained RNA was determined with a NanoDrop-2000 spectrometer. Absorbance ratios were monitored as a measure of RNA purity. The 260/280 ratio of the samples ranged between 1.95 and 2.1 and the 260/230 ratio between 2.1 and 2.25 and therefore meet the criteria for pure RNA [49].

Reverse transcription to complementary DNA (cDNA) was performed with a QuantiTect^®^ Reverse Transcription Kit (Qiagen, Hilden, Germany). Briefly, genomic DNA (gDNA) was removed via the incubation of diluted RNA with gDNA wipe out buffer followed by reverse transcription.

Gene-specific cDNA amplification was performed with a StepOnePlus™ System (Applied Biosystems, Waltham, MA, USA) using QuantiTect^®^ SYBR^®^ Green Master Mix (Qiagen, Hilden, Germany) and gene specific primers (QuantiTect^®^ Primer Assays, Qiagen, Hilden, Germany). Primer assays used in the analysis are depicted in Table 1.

In accordance with the manufacturer’s recommendations, a universal PCR protocol was used (enzyme activation: 15 min at 95 °C; 45 cycles of 15 s at 94 °C, 30 s at 55 °C and 30 s at 72 °C, followed by melting curve analysis: 15 s at 95 °C, 1 min at 60 °C, in 0.5 °C steps to 94 °C for 15 s). The melting temperature of all analyzed genes was routinely monitored and proofed to be the same within one gene, therefore indicating the same product.

Data collected with the StepOnePlus^®^ software v2.1 (Applied Biosystems, Waltham, MA, USA) were related to the mean transcript levels of the two endogenous control genes, *HPRT1* and *ALAS1*. Subsequently, the 2^−ΔΔCt^ method [31] was applied to quantify the results. As a requirement for the use of the 2^−ΔΔCt^ method, similar PCR efficiency is necessary [50] which was determined for all target and control genes and ranged between 2.1 and 2.2 with a relative standard deviation of 2%. Results are depicted as fold-changes in comparison to the respective solvent control sample, which was set to 1.

### 5.8. Immunofluorescence Analysis of LC3

The protein expression of LC3, encoded by *MAP1LC3B,* was analyzed by an immunofluorescence staining followed by microscopy/image analysis as previously described [27]. Hence, 30,000 HepG2 cells per well were seeded into µ-Slide 8 Well ibiTreat (ibidi, Gräfelfing, Germany). After a growth period of two days, cells were incubated with 0.1 or 1 ng/mL CER, 0.01 or 0.1 µg/mL DON or a respective combination of both toxins for 24 h.

Subsequently, the cells were washed once with phosphate buffered saline (PBS) followed by fixation with 3.7% formaldehyde (FA) in PBS for 15 min. Afterwards, two washing steps with PBS were performed and cells were permeabilized with 0.2% Triton X-100 in PBS for 10 min. After rinsing with PBS, blocking was performed with 1% donkey serum in PBS for one hour, directly followed by the incubation with the primary antibodies (LC3B Antibody Kit for Autophagy, Invitrogen, Thermo Fisher Scientific, Waltham, MA, USA, 1:500) for 2 h followed by three 10-min washing steps with wash buffer (0.05% Triton X-100 in PBS) and rinsing with PBS twice. Subsequently, cells were incubated with fluorescently labelled secondary antibody (donkey anti-rabbit IgG, Alexa Fluor 568, A10042 from Invitrogen, Thermo Fisher Scientific, Waltham, MA, USA; 1:1000) as well as phalloidin (Oregon Gree^®^ 488 phalloidin, O7466, from molecular probes, Eugene, OR, USA; 1:500) to stain the cytoskeletal actin filaments for 1.5 h followed by the same washing procedure as before.

Post-staining fixation was performed with 3.7% FA in PBS for 10 min and reactive sites were masked with 100 mM glycine in PBS for 5 min. The wells were treated with ROTI^®^Mount FluorCare DAPI mounting medium (Carl Roth, Karlsruhe, Germany). Pictures were acquired with an LSM Zeiss 710 microscope (Zeiss, Oberkochen, Germany) equipped with an ELYRA PS.1 system (Zeiss, Oberkochen, Germany) with an AndoriXon 897 (EMCCD) camera (Oxford Instruments, Abingdon, UK) and a Plan Apochromat 100× (1.46 NA) objective (Zeiss, Oberkochen, Germany). ZEN 2012 SP3 (black) software (Zeiss, Oberkochen, Germany) was used for image analysis and Microsoft Excel 2016 (Microsoft, Redmond, WA, USA) for further data evaluation. Experimental data includes three biological replicates and four randomly chosen optical fields per incubation condition resulting in at least 11 images/data point.

### 5.9. Statistical Analysis and Mathematical Modelling

All data was statistically analyzed and graphically depicted with OriginPro 2018G (OriginLab Corporation, Northampton, MA, USA). The Nalimov outlier test and the Shapiro-Wilk normality test were performed to eliminate outliers and test for normal distribution of the data. One sample Student’s *t*-test was performed to evaluate significant differences between the test conditions and the respective solvent control. To analyze differences between a single substance and the respective mixtures or between the measured and calculated combined effect, two-sample Student’s *t*-test was performed. One-way ANOVA followed by Bonferroni post-hoc test was performed to detect differences between the different concentrations of one substance (*p* < 0.05). The following *p* values were applied in all statistical analyses: * *p* < 0.05; ** *p* < 0.01; *** *p* < 0.001.

Mathematical modelling to predict synergism and antagonism was only possible for cell viability assays. It was performed by the calculation of expected combined effects f(ab) from the effects of the single compounds f(a) and f(b) via the “Independent Joint Action” also called ”Bliss Independence” [51,52,53,54] equation,

f(ab) = f(a) + f(b) − f(a) × f(b)(1)

The model is commonly used to calculate possible interactions [54,55,56] and assumes independent effects of the tested compounds, meaning that they have different modes of action [51] which we assume for CER and DON.

Other available mathematical models such as the “Combination Index Theorem” could not be applied as the data did not meet the criteria [51].

## Figures and Tables

**Table 1:** Specifications of investigated genes.

Protein Name	Gene Name	Gene Globe ID
Hypoxanthine phosphoribosyltransferase 1	*HPRT1*	QT00059066
Aminolevulinate synthase 1	*ALAS1*	QT00073122
Lysosome-associated membrane glycoprotein 2	*LAMP2*	QT00077063
Cathepsin D	*CTSD*	QT00020391
Microtubule-associated proteins 1A/1B light chain 3B	*MAP1LC3B*	QT00055069
Sequestosome-1	*SQSTM1*	QT00095676
Autophagy-related protein 16-1	*ATG16L1*	QT00085442

## Data Availability

Data is contained within the article.

## Supplementary Materials

The following are available online at https://www.mdpi.com/article/10.3390/toxins14020151/s1, Figure S1: Representative images of mitochondria of HepG2 cells labelled with MitoTracker® Green FM after 24 h incubation with 50 ng/mL CER, 5 µg/mL DON or the respective combination. Merge images show mitochondria and nuclei stained with MitoTracker® Green FM and Hoechst 33258, respectively. SC refers to solvent control.
