# Supplementary material for: Cereulide and Deoxynivalenol Increase LC3 Protein Levels in HepG2 Liver Cells
Source: Toxins (Basel). 2022 Feb 18;14(2):151. doi: 10.3390/toxins14020151 (PMC8880806; doi:10.3390/toxins14020151)
Supplement: Supplementary file 1 [file toxins-14-00151-s001.zip › toxins-1595728-supplementary.pdf]

toxins

MDPI

Supplementary Materials

Cereulide and Deoxynivalenol Increase LC3 Protein Levels in HepG2 Liver Cells

Julia Beisl, Gudrun Pahlke, Monika Ehling-Schulz, Giorgia Del Favero and Doris Marko

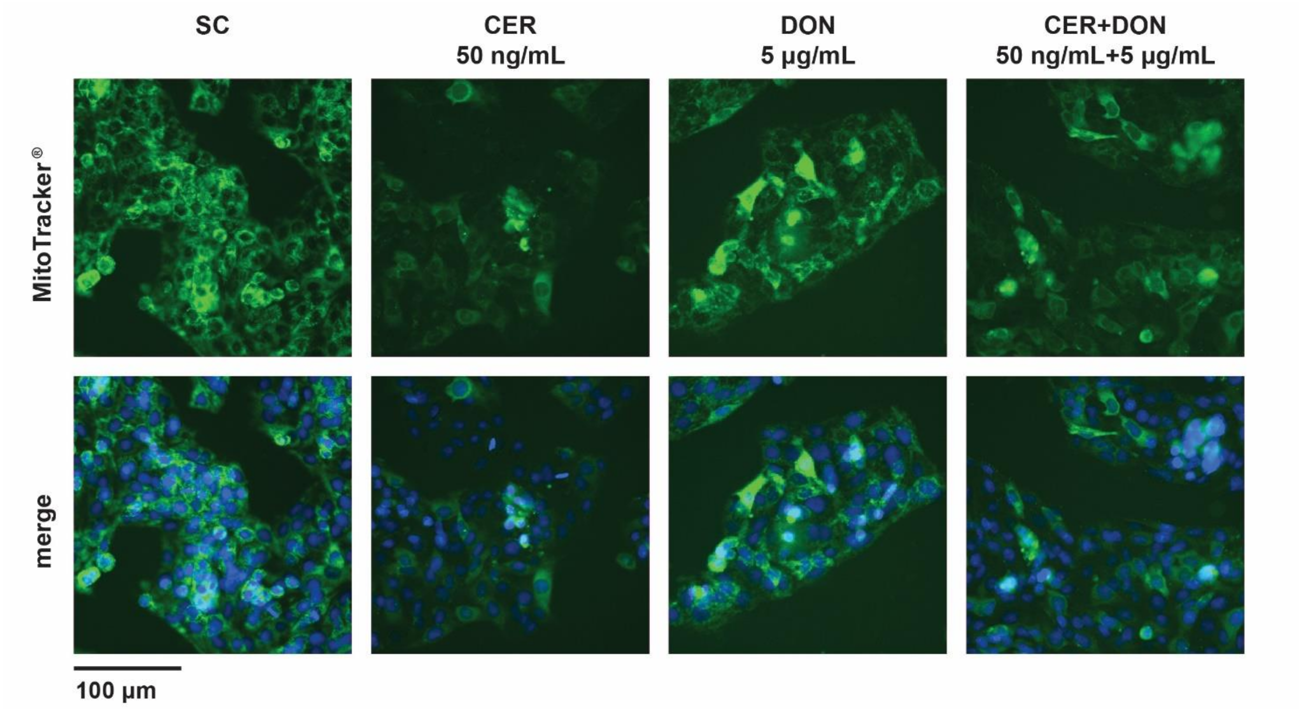

Figure S1. Representative images of mitochondria of HepG2 cells labelled with MitoTracker® Green FM after 24 h incubation with 50 ng/mL CER, 5 µg/mL DON or the respective combination. Merge images show mitochondria and nuclei stained with MitoTracker® Green FM and Hoechst 33258, respectively. SC refers to solvent control.
